# Organizing pneumonia following Covid19 pneumonia

**DOI:** 10.1007/s00508-021-01852-9

**Published:** 2021-04-16

**Authors:** Georg-Christian Funk, Caroline Nell, Wolfgang Pokieser, Birgit Thaler, Gernot Rainer, Arschang Valipour

**Affiliations:** 1 2nd Medical Department with Pneumology, Klinik Ottakring, Montleartstraße 37, 1160 Vienna, Austria; 2 Karl-Landsteiner-Institute for Lung Research and Pulmonary Oncology, Montleartstraße 37, 1160 Vienna, Austria; 3 Medical University of Vienna, Vienna, Austria; 4 Department of Pathology, Klinik Ottakring, Montleartstraße 37, 1160 Vienna, Austria; 5 IMED 19, Billrothstraße 49, 1190 Vienna, Austria; 6 Department of Respiratory and Critical Care Medicine, Klinik Floridsdorf, Brünnerstraße 68, 1210 Vienna, Austria

**Keywords:** Interstitial lung disease, Fibrosis, Long Covid, Inflammatory lung disease, Pulmonary infection

## Abstract

The potential mid-term and long-term consequences after severe acute respiratory syndrome coronavirus 2 (SARS-CoV-2) infections are as yet unknown. This is the first report of bronchoscopically verified organizing pneumonia as a complication of coronavirus disease 2019 (Covid19). It caused persisting dyspnea, impaired pulmonary function, and radiological abnormalities over 5 weeks after onset of symptoms. While organizing pneumonia frequently requires treatment with systemic corticosteroids, in this case it resolved spontaneously without treatment after 6 weeks. Healthcare professionals should consider organizing pneumonia in patients with persisting respiratory symptoms after Covid19.

## Background

The ongoing Covid-19 pandemic causes a huge burden to healthcare providers worldwide [1, 2]. The potential mid-term and long-term clinical consequences for patients after Covid-19 infections are as yet unknown. Radiological studies and clinical courses have indicated possible organizing pneumonia as a consequence of Covid19 pneumonia [3, 4]. Establishing a histological diagnosis of suspected organizing pneumonia is important as it frequently requires treatment with systemic corticosteroids [5]. We report one of the first case of histologically verified organizing pneumonia in a patient with Covid19 pneumonia.

## Case description

A 49-year-old previously healthy man had fever up to 39 °C and a dry cough starting on 22 March 2020. Prior to that a family member had experienced similar symptoms after spending time in northern Italy, which was considered the European SARS-CoV‑2 hotspot at that time. A pharyngeal swab for PCR was not obtained since he did not fulfil the case definition during that point of time. His family physician treated him with penicillin.

**Fig. 1:**
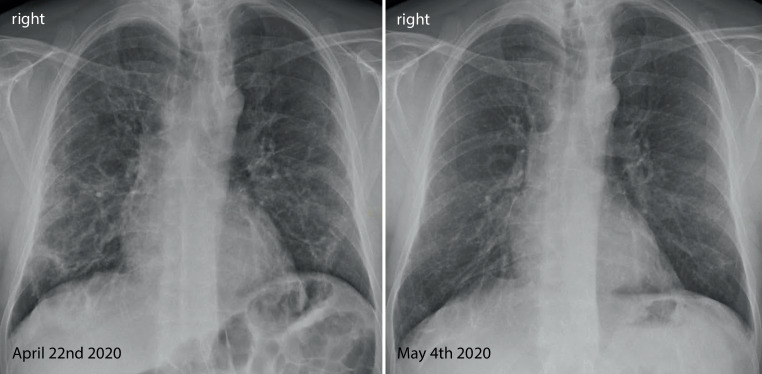
Posterior-anterior Chest X‑ray from 22 April and 4 May showing substantial spontaneous improvement of the interstitial opacities and reticular densities

**Fig. 2:**
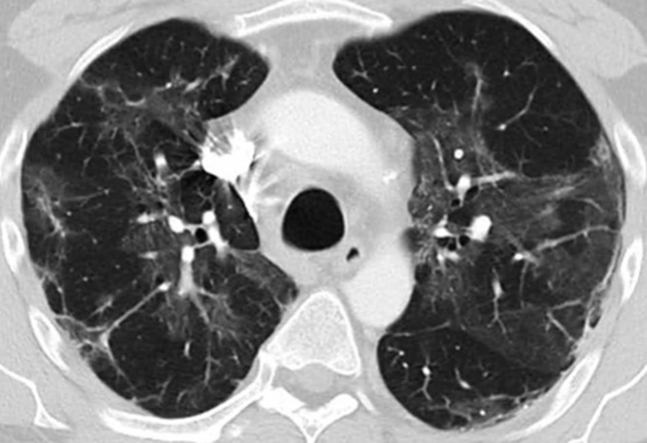
Lung CT scan in the upper lobe 4 weeks after symptom onset showing patchy subpleural ground glass opacities and linear consolidation

**Fig. 3:**
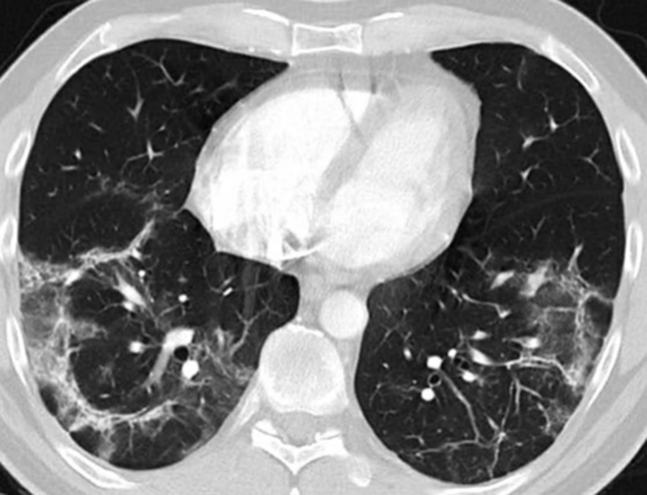
Lung CT scan in the lower lobes showing ground glass, arcade-like bands of parenchymal consolidation, peribronchial consolidation and mild bronchiolectasis

His fever disappeared but he remained substantially short of breath during minor exertion. Suspecting Covid19 he saw a pulmonologist on 15 April. Physical findings were unremarkable and the chest X‑ray showed interstitial opacities with subpleural reticular densities predominantly in the lower fields (Fig. 1). On 20 April the computed tomography (CT) scan showed subpleural patchy ground glass opacities predominantly in the upper lobes (Fig. 2). The lower lobes in addition to ground glass showed arcade-like bands of parenchymal consolidation, peribronchial consolidation and mild bronchiolectasis (Fig. 3). The CT pattern was suggestive of organizing pneumonia. He was referred to the pneumology department for further diagnostic work-up.

On 22 April pulmonary function tests showed a borderline restrictive ventilatory defect (total lung capacity 82%) without evidence of airflow obstruction. Diffusion capacity of the lung for carbon monoxide (DLCO) was reduced (59%) and blood gases showed mild hypoxemia with substantial hypocapnia (arterial partial pressure of oxygen [paO_2_] 65 mm Hg, arterial partial pressure of carbon dioxide [paCO_2_] 28 mm Hg, alveolararterial difference in partial pressure of oxygen [AaDO_2_] 51 mm Hg). Blood tests were normal apart from mildly elevated alanine aminotransferase and gamma-glutamyl transferase. The SARS-CoV‑2 PCR from a nasopharyngeal swab was negative. Serum neutralizing antibodies against SARS-CoV‑2 were positive proving the suspected Covid19 infection.

**Fig. 4:**
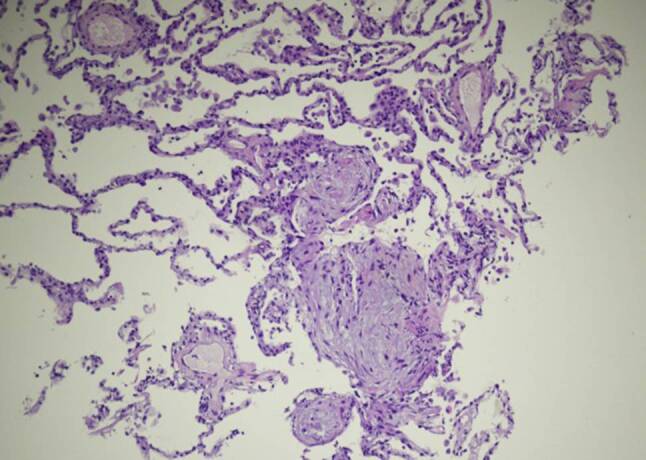
Lung histology demonstrating micropolypoid buds of pale, myxoid granulation tissue in the alveoli. These granulation areas are known as Masson bodies, protruding into the alveoli and bronchioles (hematoxylin and eosin × 100)

On 27 April bronchoscopy was performed with the patient under general anesthesia. Endobronchial findings were normal. Bronchoalveolar lavage from the middle lobe showed 41% alveolar macrophages and 59% lymphocytes. T cells were minimally elevated and the CD4/CD8 ratio was normal. Activated T cells and natural-killer-like T cells were substantially elevated (19% and 25% of lymphocytes, respectively). Bacterial culture and SARS-CoV‑2 PCR from the lavage were negative. Cytology obtained by endobronchial ultrasound-guided biopsy of a mildly enlarged subcarinal lymph node showed normal lymphocytes. Lung histology obtained by fluoroscopy-guided transbronchial biopsy from the right lower lobe demonstrated granulation tissue in the alveoli and bronchioles, typical of organizing pneumonia (Fig. 4).

At the follow-up visit on 4 May originally intended to discuss potential treatment options following histological verification of organizing pneumonia, the patient reported substantial spontaneous improvements in well-being and dyspnea, prompting repeated functional assessments. Compared to previous testing lung volumes were normalized (TLC 98%) and gas exchange improved (DLCO 70%, paO_2_ 81 mm Hg, paCO_2_ 29 mm Hg, AaDO_2_ 33 mm Hg). Chest X‑ray findings were also substantially improved (Fig. 1). Given the patients current clinical and functional status consensual agreement was made not to treat with systemic corticosteroids. Follow-up by chest X‑ray and pulmonary function tests were scheduled. The final diagnosis was organizing pneumonia following Covid19.

## Discussion

Radiological changes during Covid19 pneumonia peak around 10 days after onset of symptoms and gradually decrease thereafter [6–8]. Ground glass opacities are predominant upon onset of symptoms and progressively transform into multifocal consolidation with septal thickening [9–11]. This radiomorphological course is indicative for an evolution towards organizing pneumonia, which is a common response to lung injury [12].

Organizing pneumonia is characterized by proliferation of granulation tissue in the alveoli or alveolar ducts, with or without obliteration of distal bronchioles [5]. It can occur without an apparent cause (cryptogenic organizing pneumonia) or as a consequence of viral infections including influenza, severe acute respiratory syndrome coronavirus 1 (CoV-1) and Middle East respiratory syndrome [13–20] as well and many other underlying causes [5]. Clinical and radiological features point towards the diagnosis, which is verified by surgical or transbronchial lung biopsy [21, 22]. The clinical and radiological course of organizing pneumonia is highly variable, ranging from mild with spontaneous remission to progressive and relapsing [22]. Patients with organizing pneumonia frequently require treatment with systemic corticosteroids. So far organizing pneumonia following Covid19 has been suspected on a radiological basis and has been found in post-mortem studies [23, 24]. One case report described organizing pneumonia following Covid19 diagnosed by thoracoscopic lung biopsy [25]. To our knowledge this is the first short report of bronchoscopically verified organizing pneumonia following Covid19.

Prognosis and response to treatment in organizing pneumonia following Covid19 is so far unknown. Since corticosteroid use might be associated with increased mortality in patients with acute coronavirus pneumonia [26] histological verification of suspected organizing pneumonia seems mandatory.

## Conclusion

This is one of the first reports of organizing pneumonia as a complication of Covid19. It was the cause of persisting dyspnea, impaired pulmonary function, and radiological abnormalities 5 weeks after onset of symptoms. It improved spontaneously without treatment 6 weeks after the first symptoms. Healthcare professionals should consider organizing pneumonia in patients with persisting respiratory symptoms after Covid19.
